# Consumer Preferences and Attitudes towards Antibiotic Use in Food Animals

**DOI:** 10.3390/antibiotics12101545

**Published:** 2023-10-16

**Authors:** Katherine E. Adam, Ann Bruce

**Affiliations:** 1 Science, Technology and Innovation Studies, The University of Edinburgh, Edinburgh EH1 1LZ, UK; kadam3@ed.ac.uk; 2 Global Academy of Agriculture and Food Systems, Royal (Dick) School of Veterinary Studies, The University of Edinburgh, Roslin EH25 9RG, UK

**Keywords:** antimicrobial resistance, consumer perceptions, consumer attitudes, antibiotic use in livestock production

## Abstract

Antimicrobial resistance (AMR) is one of the major challenges faced by society, with the real threat of the failure of many medical procedures. Antibiotics are also used in livestock production and provide a potential pathway to increasing AMR. The central challenge involves ensuring animal health and welfare while securing the long-term effectiveness of antibiotics. This paper reports the results of a survey of 5693 respondents from the customer panels of four major UK supermarkets regarding preferences and attitudes towards antibiotic use in food animals, and their perspectives on how the balance between animal welfare and human benefit can be achieved. The results of these surveys are consistent with those from other countries that found that consumers generally have limited knowledge about antibiotic use in agriculture and AMR, with around 50% responding “don’t know” to many questions. There was agreement about the benefits of antibiotics outweighing harm, with 40% agreeing that, overall, the use of antibiotics to treat disease in farm animals delivers more benefit than harm. However, 44% neither agreed nor disagreed, indicating a high level of uncertainty and a situation that is potentially unstable. The seriousness of the AMR challenge is such that continued action for the more discriminating use of antibiotics must continue.

## 1. Introduction

The development of antimicrobial resistance (AMR) and the consequent prospect of future limits to medical treatments was highlighted by England’s Chief Medical Officer, Professor Dame Sally Davies, who described it as a ticking time bomb that could prevent healthcare such as cancer treatments and organ transplants in the future [1]. AMR continues to be a major public health concern: there were 178 AMR infections diagnosed per day in England in 2019, an increase of 8 per day from 2018 [2]. Although the rise in treatment-resistant infections in humans may be driven primarily by the use of antibiotics in hospital/healthcare settings [3], antibiotic use in livestock can also contribute to the development of bacterial resistance to antibiotics [4,5,6]. The influential UK report established by the then Prime Minister as an independent review, chaired by Lord O’Neill [7], advocated reducing antibiotic use in livestock production as a part of the measures aimed at mitigating the potential development of AMR. The livestock industry in the UK has responded by substantially reducing antibiotic use in recent years, with sales of veterinary medicines for food-producing animals reducing by 55% between 2014 and 2021, although this may now be reaching a plateau [8]. Data on clinical antimicrobial resistance in animals are not really available.

Potential routes for the entry of antibiotic-resistant bacteria into the human population from animal sources are various and include direct contact with animals (including pets), some foods (e.g., raw meat) and through environmental exposure (e.g., water) [9,10]. However, these linkages are not well understood. Although the focus of research is often on transmission possibilities from meat and milk, other foods (such as prawns and fruit) may also be implicated [9]. Contact with food handlers within the food chain is another potential route of transmission, although the direction of transmission is not clear [9]. 

Hocknell et al. [11] report that UK retailers have been proactive in addressing risks from antibiotic resistance emerging from food sources. However, little is known about UK consumer knowledge and perceptions about AMR and food. For example, do consumers prioritise the necessity of antibiotic use for animal welfare, or the risk of the development of antibiotic resistance? The survey reported here is intended to fill this knowledge gap. This work was carried out under the aegis of the Food Industry Initiative on Antimicrobials (FIIA) and benefits from the large consumer base available to supermarkets. Set up in 2018, the FIIA is a collaborative network of retailers, manufacturers, processors and food service companies in the UK, seeking to support responsible antibiotic use and reduce the prospect of AMR development.

Research on consumer perspectives around AMR has been limited to date, and is reviewed in the next section. We reflect further on the findings in a discussion section and draw our findings together in a final concluding section.

## 2. Literature Review

Efforts to prevent resistance by reducing antibiotic use in livestock face a central paradox: antibiotics are a valuable tool to prevent and treat animal disease, but their use carries a risk of the development of bacterial resistance. The objective of responsible antimicrobial use, as reflected in the aspirations of the FIIA and others in the food chain (including veterinarians and farmers), is to minimise usage while safeguarding animal health. 

Much of the work around behavioural change to reduce antibiotic use has focused on decisions on the use of antibiotics by those on the front line in livestock production—veterinarians and farmers—and recognises the challenges that they face [12,13]. There is evidence that both groups are concerned about the potential for resistance due to antibiotic use in livestock production, but ultimately prioritise animal health and welfare [14]. Attitudes and knowledge vary widely between key groups, e.g., veterinarians, farmers, and the general public [15], but the reduction in antibiotic use in the UK livestock sector has been driven by voluntary agriculture and food industry initiatives [11,16]. Regulation has played a more significant role in other jurisdictions, notably in Denmark [17,18]. While veterinarians did not identify consumers as an important driving force for changing antibiotic practices [19], there is evidence that broader social, political and economic factors contribute to decisions about antibiotic use [20], and public perception may be a contributing factor. 

Studies of consumers have highlighted their lack of knowledge and widespread misconceptions about the nature of antibiotic resistance and the multitude of potential pathways to resistance [10,11,21,22]. Nevertheless, AMR is widely perceived by consumers to be a potential threat to human health, e.g., in Chile [23], China [24], the USA [25,26], Italy [26], Germany [26,27], the UK [28], Canada [27,29] and the European Union [29]. Swiss consumers were generally not aware of any risks of AMR from food, having a high degree of trust that their food was safe, although expressing some concern about food from other countries [10]; however, this qualitative research was conducted using a very small sample of 14 consumers. 

Consumers often lack understanding about why antibiotics are used in agriculture [11,25,28] and are unaware of the withdrawal periods required after treating animals with antibiotics to ensure antibiotic residues are not present in food ([11,22]). The acceptability of antibiotic use seems to depend on beliefs around the value of antibiotics to improve animal welfare. Some consumers appear to be broadly supportive of the use of antibiotics for animal welfare reasons, e.g., in Cornejo et al [23] (focus group data in Chile). But acceptance can vary with the use of the antibiotic. Meerza et al. [30] found that only 14% of US consumers found antibiotic use as a growth promoter acceptable, but over 50% found the use of antibiotics to treat and control infections acceptable. Some consumers expect that no use of antibiotics means better animal welfare, when the opposite may well be true [28,30]. Meerza et al. [30], for example, found that US respondents who believed antibiotic use reduces animal welfare were more likely to be concerned about the use of antibiotics to treat, prevent and control disease than respondents who viewed antibiotics as contributing to better animal welfare. Antibiotic use may be associated in the public mind with the rise in intensive farming. Kirchelle [31], for example, suggests antibiotics made intensive farming possible, although this has been contested [32], and so antibiotic use may be associated with poorer welfare because of the intensive nature of production. There is also a degree of scepticism about claims of responsible use; for example, Bush et al. [26] reported widespread belief among consumers (including in Germany and Italy) that antibiotics are used for growth promotion, although the practice has been banned in the EU since 2006. 

Based on a survey in Ireland [22], Regan et al. found that people tended to view antibiotics, in part, through the lens of a medical model, so that their experience of General Practitioners (doctors) prescribing antibiotics to themselves and their family, reluctantly, and only in the presence of disease, contrasts with the idea of prophylactic use in agriculture [22]. The prophylactic use of antibiotics, as is common in animal production, is therefore difficult for consumers to accept. Since the study was conducted, the EU banned the routine use of antibiotics in animals in 2022 [33].

The lack of understanding about the use of antibiotics in agriculture leads to further confusion as to what is meant if the term “antibiotic-free” is used: this can be interpreted by consumers to mean that there are no antibiotic residues [26], or that no antibiotics are used during the production process. A shift towards antibiotic-free production was perceived as preferable by consumers in the USA, but had negative impacts on the health, welfare and productivity of livestock [34], demonstrating the potential trade-off between a reduction in antibiotic use and animal health and welfare. However, consumers may be unaware of the potential negative consequences of reducing antibiotic use [27]. The antibiotic-free paradigm is not one that has been embraced by UK retailers [11,35] who have instead adopted an approach of industry-wide action for the more discriminating use of antibiotics across production systems. Hocknell et al. [11] note that although retailers are able to influence, at least to some degree, the way in which antibiotics are used on farms, they have little ability to control AMR elsewhere in the broader environment of widespread antibiotic use. 

Limited evidence exists relating specifically to UK consumer preferences on antibiotic use in livestock systems and how they perceive the conflicting demands of animal welfare and managing the spread of AMR. This study, therefore, aimed to explore UK consumers’ knowledge of and attitudes towards antibiotic use in livestock, and to assess the impact on their purchasing preferences.

## 3. Results

### 3.1. Responses

A total of 5693 completed on-line questionnaires were collected across four supermarkets: 557 from supermarket 1, 814 from supermarket 2, 1524 from supermarket 3 and 2798 from supermarket 4. These questionnaires were circulated to their respective consumer panels by the individual supermarket customer insight teams.

### 3.2. Descriptive Analysis

**Figure 1:**
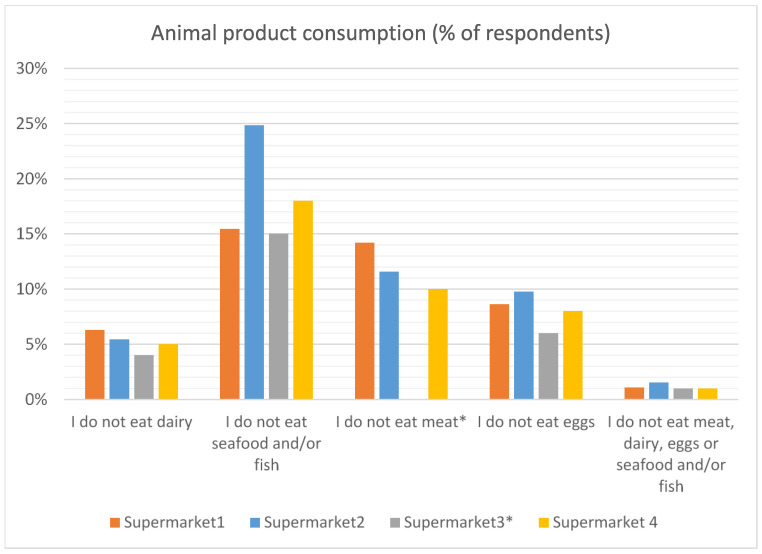
Animal product consumption. * The data from supermarket 3 presented poultry and red meat consumption separately. In total, 7% of respondents did not eat poultry and 12% did not eat red meat.

**Figure 2:**
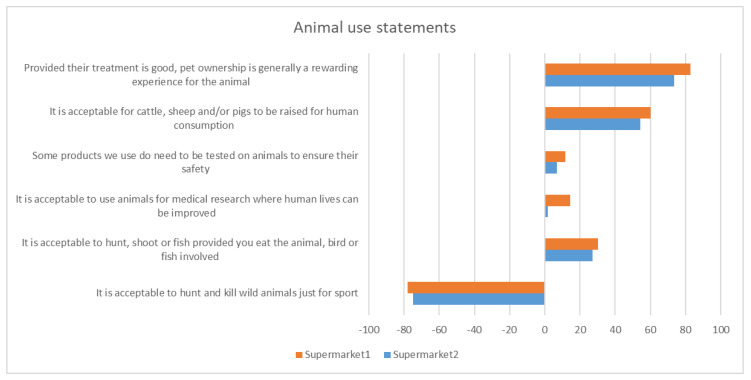
Attitudes towards animal use. X-axis presents Roselius risk ranking score for each question.

**Figure 3:**
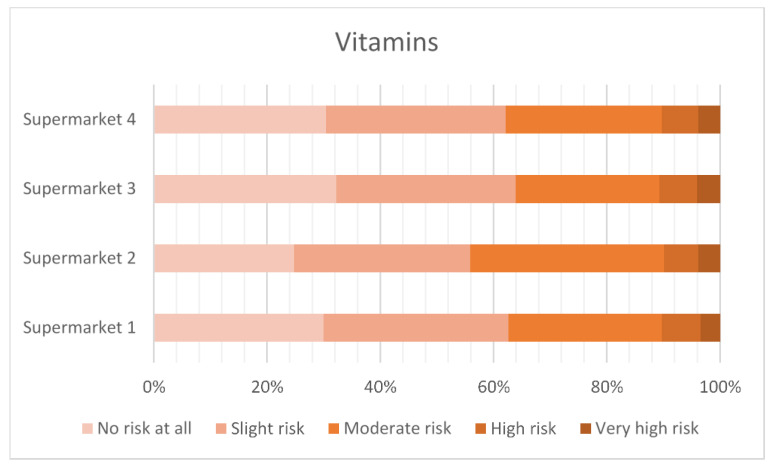
Perceived risk of vitamins.

**Figure 4:**
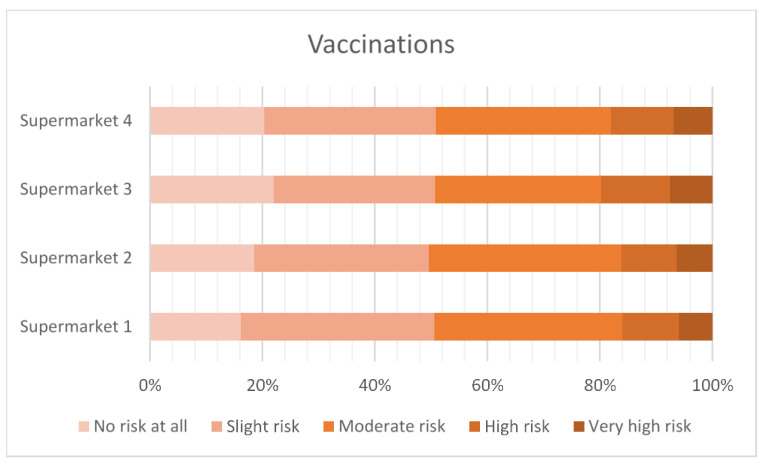
Perceived risk of vaccination.

**Figure 5:**
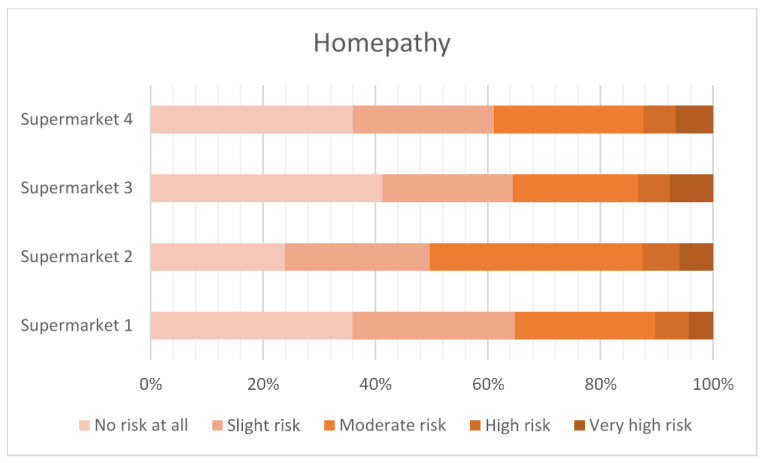
Perceived risk of homeopathy.

**Figure 6:**
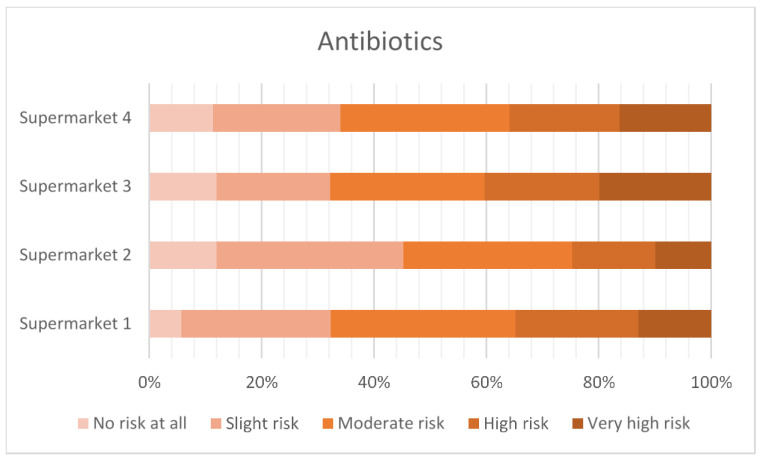
Perceived risk of antibiotics.

**Figure 7:**
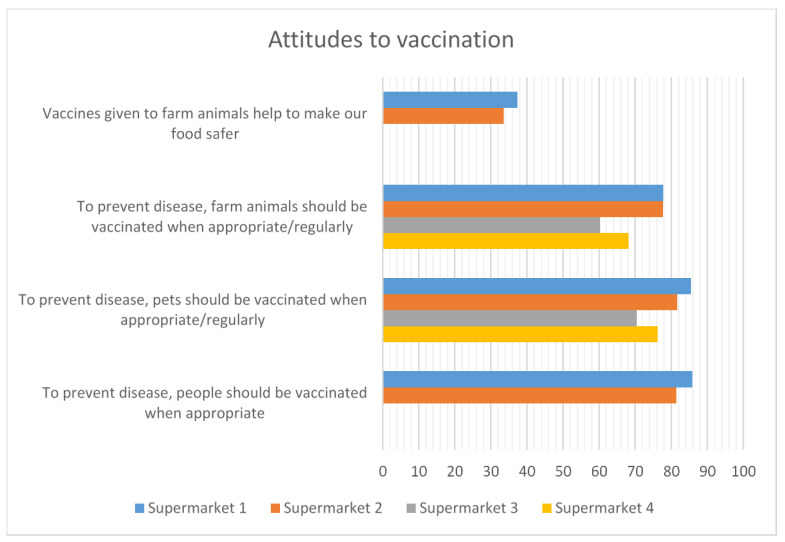
Attitudes to vaccination. Missing data for supermarkets 3 and 4 occur where questions were not included in the surveys that they conducted.

**Figure 8:**
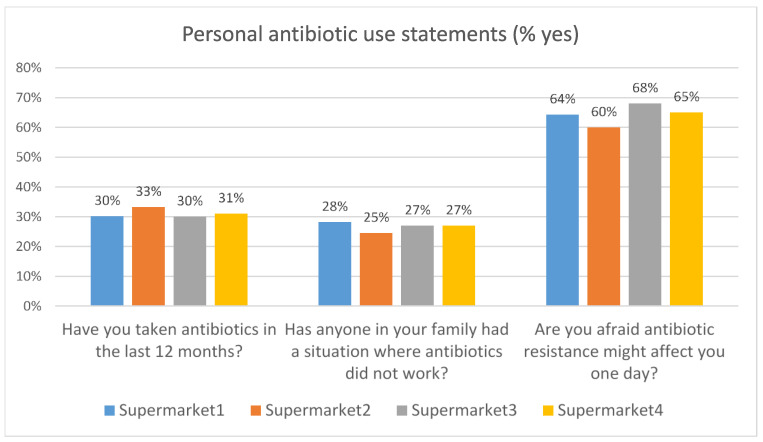
Personal experience of antibiotic use.

**Figure 9:**
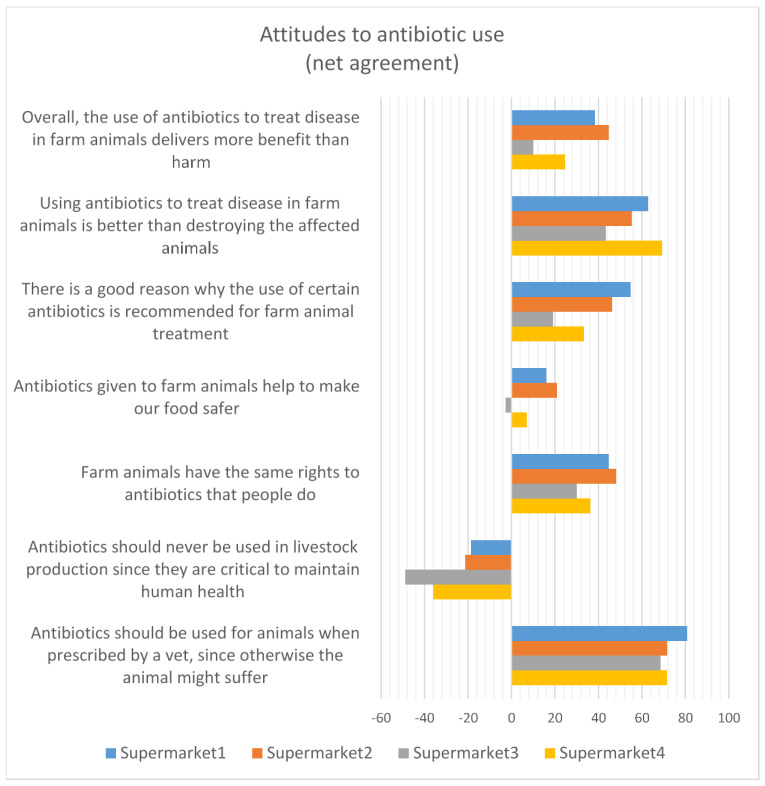
Attitudes to antibiotic use (net agreement).

**Figure 10:**
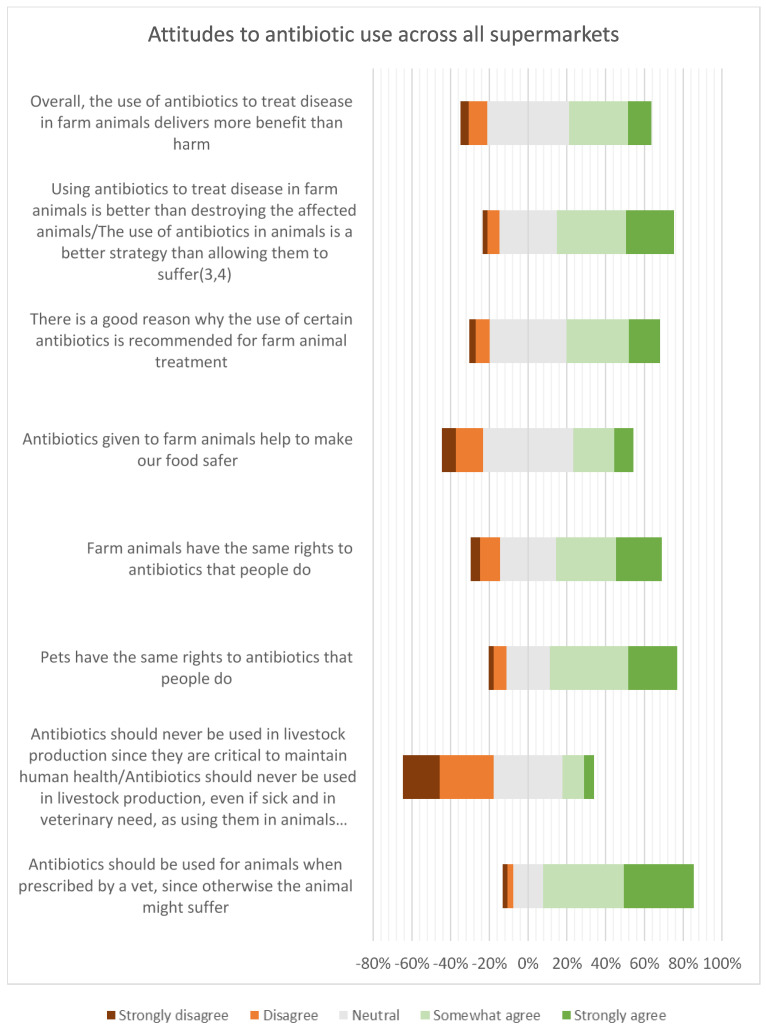
Aggregated attitudes to antibiotic use.

**Figure 11:**
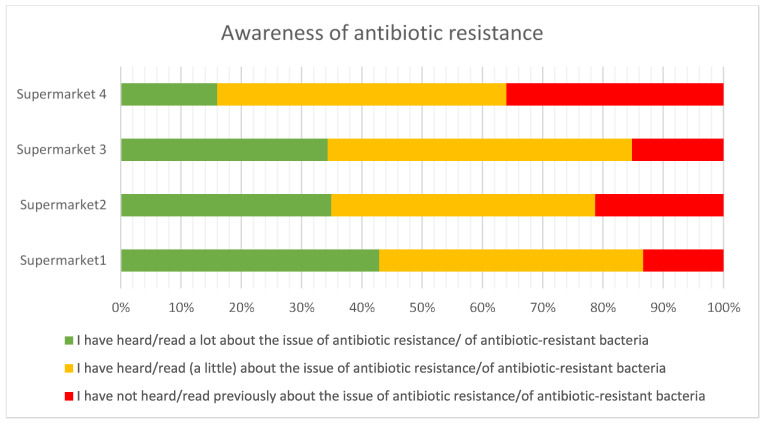
Awareness of antibiotic resistance.

**Figure 12:**
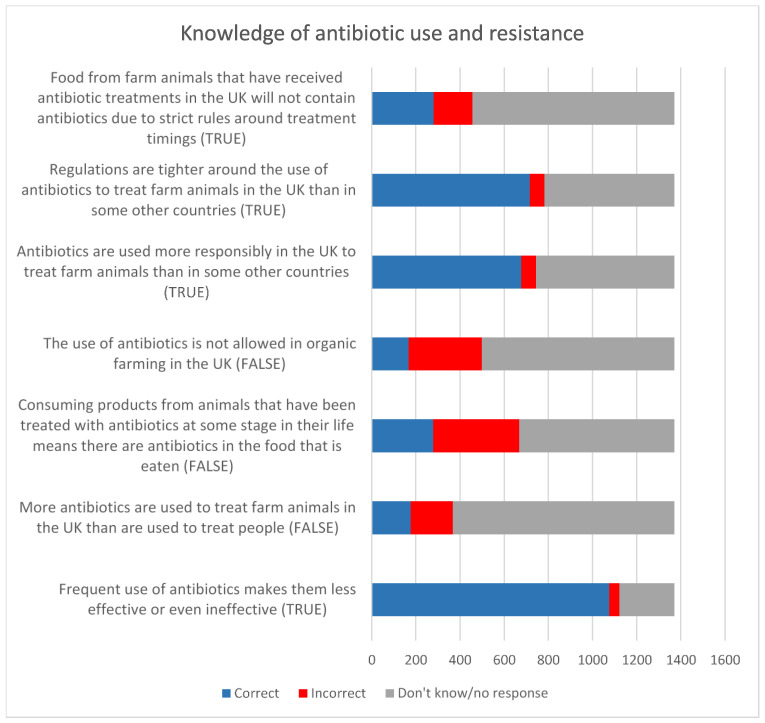
Knowledge of antibiotic use and resistance (supermarkets 1 and 2).

The following section presents the results of the descriptive analysis from all four supermarkets. Visualisations of the data for each of the main topics of interest are presented in Figure 1, Figure 2, Figure 3, Figure 4, Figure 5, Figure 6, Figure 7, Figure 8, Figure 9, Figure 10, Figure 11 and Figure 12 to provide an overview of the respondents’ attitudes, perceptions and experiences in relation to antibiotic use and resistance in livestock. No further statistical analysis for differences between supermarkets was performed on the data across all supermarkets due to data limitations and a high level of consistency in the patterns of the responses between supermarkets. The results presented in this section also describe some of the predictor variables used for the more in-depth analysis of the factors influencing consumer preferences and attitudes around antibiotic use using the more detailed data from supermarket 2.

#### 3.2.1. Animal Product Consumption

The majority of the respondents reported that they eat all animal products (Figure 1). Seafood/fish was the most commonly avoided by consumers. The number of consumers self-reporting that they do not eat meat was higher (at 10–14%) than those reported by Goddard et al. [26] at 8% in Canada and 4.8% in Germany.

#### 3.2.2. Attitudes towards Animal Use

Only supermarkets 1 and 2 asked about general attitudes towards animal use. These questions were included to gauge the respondents’ concern for animal welfare and engagement with ethical issues around the use of animals. The results (Figure 2) indicate overall agreement with the use of animals for human benefit, with the exception of hunting for sport. The levels of agreement were strongest for keeping pets and raising animals for human consumption.

#### 3.2.3. Perceived Risk of Animal Treatments

Figure 3, Figure 4, Figure 5 and Figure 6 show the level of risk perceived for vitamins, vaccinations, homeopathy and antibiotics. The majority of consumers perceived there to be some degree of risk with all animal treatments. Antibiotics were perceived to be slightly riskier than vaccination, vitamins or homeopathy.

Using vitamins, vaccines and homeopathy as comparators to the perceived riskiness of antibiotic use in food animals suggests that any intervention is seen as potentially risky by some respondents. The least risky is homeopathy, with around 35% of the respondents rating this as high or very high risk, compared to vitamins at 40%, vaccines at 50% and antibiotics at 70%.

#### 3.2.4. Attitudes to Vaccination

There was overall agreement with the vaccination of both people and animals among the respondents (Figure 7). Supermarkets 3 and 4 excluded questions about human vaccination and its impact on food safety from the surveys they distributed. The slightly lower agreement levels from supermarkets 3 and 4 may be due to the variation in the wording of the questions: supermarkets 1 and 2 worded the question as “To prevent disease, pets/farm animals should be vaccinated when appropriate”, suggesting more of a needs-based, responsive approach to vaccination, while supermarkets 3 and 4 used “To prevent disease, pets/farm animals should be vaccinated regularly”, which may have been interpreted as more of a standardised approach to vaccination.

Vaccination to prevent disease is a key alternative to the use of antibiotics once disease has been established. These data suggest that around 70% of the respondents found the appropriate or regular vaccination of farm animals acceptable. The level of acceptability is only a little less than for vaccinating pets or people. However, vaccination for food safety reasons was only acceptable to around 35% of the respondents. This may be because they are unfamiliar with the use of vaccines to control diseases in animals that can cause human health issues, although such vaccines are widely used, e.g., salmonella vaccination of laying hens [36].

#### 3.2.5. Personal Experience of Antibiotic Use

Consumers’ personal experiences of antibiotic use were similar across all four supermarkets (Figure 8). Around one in three respondents had taken antibiotics in the last year and approximately one in four had experienced antibiotic treatment failure within their family. This may not necessarily represent clinical antibiotic resistance, as treatment can fail for other reasons, but could create a heightened sensitivity to issues around antibiotic resistance among those who had had this experience. Approximately two-thirds of the respondents were concerned about being affected personally by antibiotic resistance. 

#### 3.2.6. Attitudes to Antibiotic Use in Animals

The respondents were broadly in agreement with the use of antibiotics in livestock to protect health and welfare, and disagreed overall that antibiotics should never be used in animals to protect human health (Figure 9). The overall pattern of agreement or disagreement with each of the statements presented in the questionnaire was generally consistent across all four supermarkets. The supermarkets chose to alter the wording of some questions slightly before collecting customer responses, which may have contributed to the differences observed in the level of agreement.

The aggregated responses across all four supermarkets are presented in Figure 10. This provides additional information to complement the Roselius risk rankings presented in Figure 9 for each of the supermarkets: as well as the overall levels of agreement with the statements presented to the respondents, it shows the proportion of neutral responses, shown in grey in Figure 10. The number of neutral responses was relatively high, potentially indicating either uncertainty or a lack of concern about antibiotic use in animals. It is worth noting that the results also reflect an inherent uncertainty: 28% of respondents from one of the supermarkets agreed with both statements “antibiotics should never be used” and “should be used when necessary”.

#### 3.2.7. Awareness of Antibiotic Resistance

Figure 11 demonstrates that the majority of consumers have some level of awareness of antibiotic resistance, but there was some variation between supermarkets—as an example, the proportion of respondents reporting high levels of awareness of antibiotic resistance varied from 43% in supermarket 1 to 16% in supermarket 4. The proportion of respondents reporting no awareness of AMR was perhaps surprisingly high at 36% in supermarket 4, given the attention that the topic has received in the media in recent years.

#### 3.2.8. Knowledge of Antibiotic Use and Resistance

Only supermarkets 1 and 2 collected data on consumers’ knowledge about antibiotic use and resistance in farmed animals. The results are presented in Figure 12 to show the proportion of correct answers, indicating some level of knowledge, and incorrect answers, indicating a misperception, and “don’t know”, indicating a lack of knowledge, in response to “true or false” statements relating to the topic. The pattern of responses was highly consistent across the two supermarkets. Consumers’ knowledge was strongest around the potential for frequent use of antibiotics to lead to resistance, and that the UK has higher standards around antibiotic use in livestock than some other countries. Knowledge was weaker around antibiotic residues in animal products, antibiotic use in organic production and the relative quantities of antibiotics used in people and animals. The proportion of “don’t know” answers was high, at over 50% for most questions, indicating that consumers generally lack knowledge of these topics. 

### 3.3. Inferential Analysis

Further analysis of the data was conducted to build on the descriptive results presented in order to explore the association of various factors with (1) consumer attitudes towards antibiotic use and (2) consumers’ purchasing preferences. Only supermarket 2 provided sufficiently granular data for this more in-depth analysis.

#### 3.3.1. Demographics

Supermarket 2 collected basic demographic information from respondents relating to gender, age, location and animal ownership. Table 1 presents an overview of the demographics of the respondents from supermarket 2 in comparison to the UK population, suggesting that the respondents are broadly representative of the population as a whole, although women, over-55s and rural dwellers are slightly overrepresented—perhaps reflecting the population of supermarket shoppers, and those with an interest in agricultural topics. The source of the data for the UK population is indicated below the table.

#### 3.3.2. Consumer Attitudes towards Antibiotic Use in Livestock

Consumer attitudes, as represented by agreement with the statement “Overall, the use of antibiotics to treat disease in farm animals delivers more benefit than harm” from 767 respondents from supermarket 2, were divided into those who agreed (n = 397), disagreed (n = 54) and neither agreed nor disagreed (neutral) (n = 316). As shown across all supermarkets in Figure 9 and Figure 10, the majority agreed that the benefits of antibiotic use in farm animals outweigh the harm.

The results of the univariable analysis (Table 2) identified a statistically significant association (*p* < 0.05) between attitudes towards antibiotic use in livestock and keeping animals (*p* = 0.016), consuming animal foods (*p* = 0.038), agreement with vaccination (*p* < 0.001), agreement with animal use (*p* = 0.003), knowledge of antibiotic resistance (*p* < 0.001), having used antibiotics in the last 12 months (*p* < 0.001) and the perceived risk of antibiotic use in livestock (*p* < 0.001). Gender, age, concern for animal welfare, level of concern about antibiotic resistance, fear of being affected by antibiotic resistance, perceived importance of antibiotic resistance, experience of antibiotic treatment failure and awareness of antibiotic resistance were not significantly associated with this outcome.

The results of the multivariable original logistic regression model are presented in Table 3. When all of the relevant predictor variables are considered, respondents who keep animals, are in favour of vaccine use, have greater knowledge of antibiotic use and resistance, and have taken antibiotics in the last year were more likely to agree that the benefits of antibiotic treatment in farm animals outweigh the harm. The respondents who perceived that the use of antibiotics in animals represented a higher level of risk to the consumer were less likely to agree that the benefits outweighed the harm.

#### 3.3.3. Consumer Preferences for Products Labelled Antibiotic-Free

The analysis was repeated with “Preference for antibiotic-free animal products” as the outcome, based on agreement with the statement “If there was a label showing food came from an animal that had never had antibiotic treatments, I would choose it preferentially”. Of the 750 responses, 133 disagreed with the statement, 240 agreed and 377 neither agreed nor disagreed. The high proportion of neutral responses indicates a high level of uncertainty around this issue. 

The results from the univariable analysis (Table 4) showed that being female (*p* = 0.036), aged over 55 (*p* = 0.004), knowledge of antibiotic resistance (*p* = 0.02), concern about antibiotic resistance (*p* < 0.001), fear of being affected by antibiotic resistance (*p* = 0.012), personal experience of antibiotic treatment failure (*p* = 0.036), perceived risk of antibiotic use in livestock (*p* < 0.001) and awareness of antibiotic resistance (*p* = 0.018) were statistically significantly associated with this outcome. 

The results of the ordinal logistic regression model are presented in Table 5. Female respondents were more likely to select a neutral response to whether they would prefer products produced without antibiotics, while male respondents were more likely to either agree or disagree that they would prefer products produced without antibiotics. Respondents who have had a family member affected by antibiotic treatment failure were less likely to select a neutral response (i.e., were more likely to have a definite opinion and either prefer or not prefer antibiotic-free production). Respondents with a lower level of knowledge about antibiotic use and resistance were more likely to prefer products produced without antibiotics. Respondents with a higher level of knowledge of antibiotic use and resistance were more likely to give a neutral response. The more concerned, but uncertain, that respondents felt about antibiotic resistance (agreement with the statement “I am very concerned about the issue of antibiotic resistance but not sure what I can do”), the more likely they were to prefer antibiotic-free products. The higher their perception of risk to the consumer from the use of antibiotics in the food chain, the more likely the respondents were to prefer antibiotic-free products.

## 4. Discussion

There was general agreement with the use of animals for human benefit, with around 50% agreeing that it is acceptable for cattle, sheep and/or pigs to be raised for human consumption. The number of consumers self-reporting that they do not eat meat was higher (at 10–14%) than those reported by Goddard et al. [27] at 8% in Canada and 4.8% in Germany. One possible explanation is that this is a reflection of the recent increasing emphasis on reducing meat eating to reduce climate change impacts.

Forty percent of the respondents agreed that, overall, the use of antibiotics to treat disease in farm animals delivers more benefit than harm, but 44% neither agreed nor disagreed, indicating a high level of uncertainty. Consumers who keep animals, have greater knowledge of antibiotic use and resistance, and have received antibiotic treatment in the last 12 months were more likely to agree that antibiotic use was more beneficial than harmful. However, the high proportion of neutral responses to attitudinal questions may indicate a situation that is not stable and could swing in different directions. Around 65% of consumers were concerned about being affected by AMR personally. However, the high levels of concern about animal welfare may be ameliorating this personal focus, resulting in a strong recognition that antibiotics should be available for treating animals when necessary. Around 50% of the respondents agreed that farm animals have the same rights to antibiotics as people do (this figure was very slightly lower than the rights of pets to antibiotics). Perhaps as a result, there was general lack of support for antibiotic-free production among these UK respondents. The data on concern about the use of antibiotics should also be viewed in the context of perceptions of risk from alternative interventions, such as vaccination (viewed as high or very high risk by 50% of the respondents in this survey) and even vitamins (viewed as high or very high risk by 40% of the respondents). 

The perception that UK farmers use antibiotics more responsibly than in some other countries (around 50% agreement) may result in a stronger level of trust in the food chain. This would fit with the data from Swiss consumers [10] who trusted their food sources and where antibiotic use in agriculture is low, and US consumers who preferred antibiotic-free production [25] where, until 2017, antibiotics important for human health have been permitted for use for growth promotion [30]. This is an important finding given that consumers have a vested interest in how their food is produced, while they have limited control over production systems and any risks they may inadvertently be exposed to. Food is, of course, traded internationally, and the small number of Swiss consumers interviewed [10] expressed greater concern about AMR spread from consuming food produced elsewhere in the world. 

Although there was broad acceptance for the use of antibiotics in food production among these UK respondents, a minority (7%) perceived that the use of antibiotics in animals represented a level of risk to the consumer that meant the benefits did not outweigh the harm. These consumers should not be neglected.

The results of these surveys are consistent with those from other countries that found that consumers generally have limited knowledge about antibiotic use in agriculture and AMR, with over 50% responding “don’t know” to questions about these aspects. Consumers’ knowledge was strongest around the potential for the frequent use of antibiotics to lead to resistance. The knowledge was weaker around antibiotic residues in animal products, antibiotic use in organic production and the relative quantities of antibiotics used in people and animals.

Redding et al. [37] suggested educating consumers more effectively on the processes taken to limit the occurrence of antibiotic residues in food (milk, in their specific case) to effect behavioural change, leading to reduced concern about antibiotic use. They based this conclusion on testing out the impacts of providing brochures or videos with information on antibiotic residue mitigation methods, and found that these approaches altered US consumer behaviour. However, while potentially reassuring consumers about residues, education does not impact on any principle- or value-based concerns around the use of antibiotics in livestock production. Meerza [30] further found that consumers with little knowledge about antibiotic use in animal agriculture were likely to avoid this kind of information. This approach also assumes that antibiotic residue mitigation methods are uniformly practised, and this may not always be the case.

The question as to whether food produced without the use of antibiotics should be identified by labels is one area where supermarkets could act. The data from this survey suggest a great deal of uncertainty as to whether such labelling would be desirable or not, with 32% of respondents agreeing that they would prefer food labelled as never having received antibiotic treatments, whereas 50% were neither supportive nor unsupportive of this initiative. Data from other research [38] suggest that while US consumers are generally supportive of such labels, they are less willing to pay for them. Goddard [27] found that 9% of German and 15% of Canadian respondents indicated they would be willing to pay for reduced antibiotic use, while Ding et al. [24] found that an astonishing 79.8% of Chinese respondents were willing to pay extra for products free from antibiotics. Whether such stated willingness would be repeated in making purchases remains a question. There are some initiatives to provide antibiotic-treatment-free products in the UK [28], so the success or otherwise of these in the market place will also test the desirability of labelling. An alternative, as reportedly implemented in Sweden [39], is to use on-line sustainability declarations that are available to the supermarkets, but not the consumers, in order to track the exposure of animals to antibiotics.

Another alternative would be for supermarkets to provide financial incentives to livestock producers to implement alternative strategies to reduce antimicrobial use, such as increased hygiene and biosecurity or vaccination, which may incur additional costs for the producer compared to antibiotics. However, as noted earlier, vaccination was viewed as high or very high risk by 50% of the respondents in this survey. The involvement of veterinarians may also be critical for assuring the responsible use of antibiotics. The surveys reported here found around 70% agreement that antibiotics should be used when prescribed by a veterinarian. Similarly, other research suggested that the responsible use of antibiotics was contingent on the verification of a bacterial infection by either a veterinary surgeon or a test [26]. These types of initiatives would require supermarkets to have a direct supply chain with a clear and long-term link to their suppliers, and consumers willing to pay for such measures. Hughes et al. [35] emphasised how different supply chain structures impact on the ability of retailers to influence antibiotic use in global value chains.

The focus on people as consumers rather than citizens places the emphasis and responsibility for AMR on the consumers who have the least information available to them. But people are also citizens, and if treated as such, the views of those who do not consume products from animals also become important. From the perspective of citizens, AMR will continue to be an important focus, and something that cannot be neglected. The pressure to reduce use of antibiotics in animal agriculture will continue. To quote:

“It would be a brave politician who, after having made the electorate and their children jump through inconvenient and expensive hoops before being prescribed an antibiotic, would not expect livestock and companion animals to jump even higher”

([40], p. 169).

Further research is needed on the practicalities of continuing to reduce antibiotic use and in what consumers and citizens understand as responsible use. Having a better understanding of the balance point where the benefits outweigh the risks requires an understanding of the degree of “harm” that is deemed acceptable (e.g., when an animal has accidentally harmed itself versus specific disease conditions), whether this varies between species (e.g., pigs versus beef cattle), among different production systems (extensive versus intensive) or among antibiotics with greater or lesser propensity to drive the evolution of resistance or result in environmental exposure (e.g., how the antibiotic is metabolised and excreted). 

The results from these surveys consist of a large dataset on consumer perceptions. Because each supermarket conducted their own survey, there were some variations between the datasets such as the precise wording of the questions and the level of data aggregation that presented additional challenges for data management and analysis. The authors consider that these minor issues with data quality are offset by the enhanced access to supermarket customers offered through our collaboration with commercial partners to conduct this research. As the survey started from the same questionnaire basis and the limitations in terms of data consistency are described clearly, we suggest that the findings provide a robust evidence base for further discussion about the role of the consumer in driving sustainable antibiotic use in agriculture. 

The surveys were initiated by the supermarkets through their respective consumer panels, as opposed to being conducted by external research organisations. It is possible that the answers given may have been influenced by the existing relationship that these consumers have with the supermarket in question, given that consumers in the customer insight panels are likely to have responded to previous questionnaires. However, the response rate to questionnaires conducted by external researchers is often low, and it is therefore possible that the responses from a consumer panel are more representative of the customers of that supermarket than an attempt to recruit consumers to respond to questionnaires by a totally external actor would be to the population as a whole. While responses from a single supermarket may reflect the particular demography of the customers of that supermarket, combining data from four different supermarkets would be expected to compensate for any such effect, and the responses across all four supermarkets were broadly similar. The results of the more detailed inferential analysis possible for data from supermarket 2 may not necessarily apply more generally. However, the available demographic data suggest a reasonably representative sample of the general UK population in the data from supermarket 2, and the sample size from supermarket 2 is of a size comparable with other studies on consumer attitudes towards the use of antibiotics in food production. Furthermore, many people will shop in more than one outlet, so customers of supermarket 2 may also be customers of other retail outlets.

Undertaking a survey of this size and scope from a purely academic source would be challenging to achieve, expensive and very time-consuming. The willingness of the supermarkets to collaborate pre-competitively to obtain these data demonstrates an industry-wide commitment to tackling AMR in the best ways possible. To quote Hocknell et al., “Overcoming the AMR challenge cannot become an industry competitive issue and it cannot be something the UK, or Europe, does by itself. There needs to be continued acceptance across human and animal health, and the whole food system that microbes know no borders” ([11], p. 11). This viewpoint is now more apparent due to the COVID-19 pandemic than when this statement was written. 

## 5. Materials and Methods

This research was initiated as part of an FIIA working group that sought to investigate consumers’ awareness and understanding of the use of antibiotics in livestock production and antibiotic resistance. 

### 5.1. Questionnaire Design

A questionnaire was designed by the authors in collaboration with a working group of the FIIA and in consultation with relevant supermarkets’ customer insight teams. In order to ensure comparability with existing research data (as far as was possible), it was based on the questionnaire used in previous studies in Germany and Canada [27]. The questionnaire was adapted for a UK audience by the FIIA working group and adjusted by the customer insight teams based on their expertise in surveying their customer base. This led to some minor variations in the wording and order of the questions, although this did not fundamentally change the nature of the questions presented to the respondents. Any variations in the questions between supermarkets are described in the results for each question. The questionnaire covered consumer’s consumption of animal products; attitudes towards use of animals for food, research, companionship and sport; the perceived risk of treatments administered to food animals; attitudes towards antibiotic use in animals; attitudes towards vaccination; personal experience of antibiotic use and treatment failure; awareness of the issue of antibiotic resistance; knowledge of antibiotic use and antimicrobial resistance; and preferences for antibiotic-free animal production. Basic demographic information about the respondents was also collected by one of the supermarkets. 

### 5.2. Data Collection

The questionnaire was distributed by the customer insight teams of the four participating supermarkets to customers. Some minor alterations to the questions included were made by each supermarket, but the topics covered remained broadly the same across all of the surveys. Any variations in wording or questions that were omitted are reported along with the relevant results. Each supermarket applied their own standards for sharing anonymous data; for example, the Terms and Conditions for joining the consumer panel included an agreement that general anonymised sentiment gathered through the research could be shared with appropriate parties, specifically for research purposes, and no identifying data were shared. The supermarkets that collected the data confirmed they are content with the data analysis being made available for publication. Ethics clearance on this basis was obtained from the University of Edinburgh, number 284340. Data were collected over the period August–September 2019.

### 5.3. Data Analysis

After all data had been collected, the supermarkets provided anonymised datasets to the academic researchers for further analysis. 

### 5.4. Descriptive Analysis

Preliminary descriptive analysis was performed to summarise the responses to each question from all four supermarkets. For attitudinal questions using a Likert scale to indicate the respondents’ level of agreement with various statements, the Roselius risk ranking method [41] was used to calculate the overall level of agreement or disagreement, as in previous studies of consumer attitudes and AMR [27]. The difference between the number of positive and negative responses was calculated, and then divided by the total number of responses and multiplied by 100 to provide the value of “net agreement”. Net agreement with each statement is therefore given a quantitative value between −100 (total disagreement) and 100 (total agreement).

### 5.5. Inferential Analysis

#### 5.5.1. Predictor Variables

Further inferential analysis was performed to assess the association between the variables included in the questionnaire and two outcomes: (i) consumers’ attitudes towards antibiotic use in production animals and (ii) consumers’ preferences for food produced without antibiotics. Only one of the four supermarkets provided a full dataset suitable for this more detailed analysis. Therefore, the inferential analysis was only performed for supermarket 2. The basic demographic data provided by the respondents was compared with UK population data to assess how closely the respondents represented the overall UK population. The inferential analysis was conducted using R software (v3.5.3) [42].

For the variables marked with an asterisk in Table 6, scores were created by calculating the sum of the responses from multiple relevant survey questions for each respondent based on numerical values allocated to each response. The scores were used for further analysis to minimise issues of multiple comparisons and to provide a clearer overview of the attitudes to each topic, rather than responses to an individual statement, which are more likely to be influenced by the precise wording used. For “animal products consumed” (1), a value of 1 was assigned for each category of product consumed, e.g., meat, fish/seafood or dairy. For attitudinal variables relating to vaccination (2), animal use (3) and antibiotic use in livestock (4), a numerical value was assigned to the level of agreement (strongly disagree = −2, disagree = −1, neutral = 0, agree = 1, strongly agree = 2) with positive statements. For negative statements (e.g., for attitudes to antibiotic use, “Antibiotics should never be used”), the scores assigned were reversed (i.e., strongly disagree = 2, strongly agree = −2). For “Knowledge of antibiotic resistance” (5), a value of 1 was given for each correct answer, 0 for “don’t know”, and −1 for incorrect answers. Variables relating to perceived risk were assigned values on a five-point scale from 0 (no risk) to 4 (very high risk). Variables for the perceived importance of topics (e.g., animal welfare, antibiotic resistance, sustainability) were scored from 1 (slightly important) to 4 (highly important).

#### 5.5.2. Outcome Variables

Consumer attitudes towards antibiotic use were inferred from the strength of agreement with the statement “Overall, the use of antibiotics to treat disease in farm animals delivers more benefit than harm”. This statement was selected as the outcome variable to reflect consumers’ overall assessment of the balance of benefits and harms of antibiotic use. Consumer purchasing preferences around antibiotic-free animal products in livestock production were determined by the level of agreement with the statement “If there was a label showing food came from an animal that had never had antibiotic treatments, I would choose it preferentially”. Both outcomes were analysed as ordinal variables with three levels. “Agree” comprises all positive responses (“agree” or “strongly agree”), “disagree” includes all negative responses (“disagree” or “strongly disagree”) and “neutral” consists of all responses that were neither favourable nor unfavourable. 

#### 5.5.3. Analysis

Univariable analysis was performed using ordinal logistic regression to assess the association between each predictor variable shown in Table 6 and each of the two outcome variables: (1) Consumer attitudes towards antibiotic use in livestock and (2) Consumer preferences for products labelled antibiotic-free. Two multivariable ordinal logistic regression models were then developed, one for each outcome, including only variables with a *p*-value of <0.1 and an odds ratio with a 95% confidence interval that did not include 1 in the results from the univariable analysis. Backward stepwise regression was performed to eliminate variables and produce a final model that best fit the observed data.

## 6. Conclusions

The development of AMR continues to be a threat to human health. The use of antibiotics in livestock production has the potential to contribute to the development of AMR, but efforts to prevent resistance by reducing antibiotic use in livestock face a central paradox: antibiotics are a valuable tool to prevent and treat animal disease, but their use carries a risk of the development of bacterial resistance. Little is known about UK consumer knowledge and perceptions about AMR and food. For example, do consumers prioritise the necessity of antibiotic use for animal welfare, or the risk of development of antibiotic resistance? The survey reported here is intended to fill this knowledge gap, and benefits from access to the large consumer base available to supermarkets. A survey of 5693 people from the customer insight panels of four major UK supermarkets was used to better understand consumer attitudes and perceptions. Consistent with other surveys, the data indicate that there is limited knowledge about antibiotic use and AMR among consumers. While there was a degree of consumer concern about antibiotic use in food production, it was not a major issue for the majority of the respondents. These data do not indicate a strong drive for a reduction in antibiotic use in farm animals from a consumer perspective, perhaps reflecting the recognition by consumers that the level of antibiotic use in UK agriculture is low by global standards. There was agreement about the benefits of antibiotics outweighing the harm, with 40% agreeing that, overall, the use of antibiotics to treat disease in farm animals delivers more benefit than harm. However, 44% neither agreed nor disagreed, indicating a high level of uncertainty and a situation that is potentially unstable. Combined with the low level of knowledge of antibiotic use and AMR in animal production, attitudes may change.

The study identified factors that influence consumer attitudes towards antibiotic use in agriculture, but further work would also permit a more in-depth exploration of these factors. Antibiotic-free production was not widely supported by UK consumers, particularly if it results in lower levels of health and welfare for production animals, suggesting this may not be an appropriate strategy for the UK market. However, the seriousness of the AMR challenge is such that continued action for the more discriminating use of antibiotics must continue.

## Figures and Tables

**Table 1:** Demographics of respondents and UK population.

Variable		Supermarket 2 Respondents	UK Population *
Gender ^1^	Male	41.3%	49%
	Female	58.7%	51%
Age ^2^	18–34	31%	-
	20–34	-	26%
	35–44	16.5%	16%
	45–54	17.5%	18%
	55 or over	33.8%	40%
	Prefer not to say	1.2%	-
Region ^3^	East Midlands	8.5%	7.2%
	East of England	6.0%	9.3%
	London	13.5%	13.4%
	North East	6.0%	4.0%
	North West	11.5%	11.0%
	Scotland	6.0%	8.2%
	South East	15.5%	13.7%
	South West	11.1%	8.4%
	Wales	3.8%	4.7%
	West Midlands	9.7%	8.9%
	Yorkshire and the Humber	7.5%	8.2%
	Prefer not to say	0.9%	-
Rural/urban ^4^	Rural	27.9%	16.5%
	Urban	66.3%	82.9%
	Prefer not to say	5.8%	-
Pet ownership ^5^		42.7%	40%

* UK population size = 66,796,807 (mid-2019 ONS data). ^1^ Gender: https://www.ethnicity-facts-figures.service.gov.uk/uk-population-by-ethnicity/demographics/male-and-female-populations/latest 2011 census data. ^2^ Source: Mid-2019: https://www.ons.gov.uk/peoplepopulationandcommunity/populationandmigration/populationestimates/bulletins/annualmidyearpopulationestimates/mid2019. NB: The age categories used by the government differ slightly, hence the age category of 20–34 for government data compared to the supermarket survey category of 18–34. Government data categories are 15–19 and 20–24. Denominator data for the government data percentages shown here are as the percentage of adults over 20 in the UK (51,178,858). ^3^ Source: Mid-2019: https://www.ons.gov.uk/peoplepopulationandcommunity/populationandmigration/populationestimates/bulletins/annualmidyearpopulationestimates/mid2019#population-estimates-data. Office for National Statistics, National Records of Scotland, Northern Ireland Statistics and Research Agency—Population Estimates. ^4^ https://www.statista.com/statistics/984702/urban-and-rural-population-of-the-uk/, https://databank.worldbank.org/source/population-estimates-and-projections#. NB: Same population assumed as denominator as for regional population. ^5^ https://www.statista.com/statistics/308218/leading-ten-pets-ranked-by-household-ownership-in-the-united-kingdom-uk/. All website links accessed on 14 November 2020.

**Table 2:** Univariable analysis—consumer attitudes towards antibiotic use in livestock. * = Statistically significant (*p* < 0.05).

Variable	*p*-Value	Odds Ratio (95% Confidence Interval)
Female gender	0.259	1.174 (0.889–1.552)
Age	Baseline < 35	
	Age 35–44: 0.057	1.500 (0.989–2.280)
	Age 45–54: 0.959	0.989 (0.654–1.493)
	Age 55+: 0.060	0.717 (0.506–1.013)
	Prefer not to say: 0.061	4.590 (1.082–31.240)
Urban location	0.079	0.772 (0.578–1.031)
Keeping pets/livestock *	0.016 *	0.710 (0.537–0.937)
Animal food consumed (1) *	0.038 *	0.846 (0.722–0.990)
Attitudes to vaccination (2) *	<0.001 *	0.348 (0.276–0.4351)
Attitudes to animal use (3) *	0.033 *	0.969 (0.941–0.997)
Importance of animal welfare	0.312	0.952 (0.866–1.047)
Knowledge of antibiotic resistance (5) *	<0.001 *	0.757 (0.699–0.818)
Agreement with “I am very concerned about the issue of antibiotic resistance but not sure what I can do”	0.577	1.043 (0.899–1.212)
Are you afraid that antibiotic resistance might affect you one day?—yes	0.161	1.285 (0.907–1.828)
Importance of AMR	0.889	0.992 (0.888–1.108)
Antibiotic use in last 12 months *	<0.001 *	0.557 (0.407–0.760)
Experience of AMR in family	0.681	1.076 (0.759–1.520)
Perceived risk of antibiotics *	<0.001 *	1.390 (1.238–1.565)
Awareness of AMR	0.134	0.865 (0.715–1.046)
Importance of sustainability	0.354	1.056 (0.9411.186)

**Table 3:** Results of ordinal logistic regression model for consumer attitudes.

Variable	*p*-Value	Odds Ratio (95% Confidence Interval)
Keeping pets/livestock	0.049	0.73 (0.53–1.00)
Attitudes to vaccination	<0.001	0.43 (0.34–0.55)
Knowledge of antibiotic use and resistance	0.002	0.87 (0.79–0.95)
Antibiotic use in last 12 months	<0.001	0.56 (0.40–0.78)
Perceived risk of antibiotics	<0.001	1.25 (1.10–1.44)

**Table 4:** Univariable analysis—consumer preferences for antibiotic-free products. * = Statistically significant (*p* < 0.05).

Variable	*p*-Value	Odds Ratio (95% Confidence Interval)
Female gender *	0.036 *	1.342 (1.020–1.766)
Age *	Baseline < 35	
	Age 35–44: 0.356	0.822 (0.542–1.249)
	Age 45–54: 0.531	0.878 (0.585–1.321)
	Age 55+: 0.004 *	0.610 (0.435–0.854)
	Prefer not to say: 0.125	5.238 (0.907–98.860)
Urban location	0.056	1.321 (0.993–1.755)
Keeping pets/livestock	0.493	1.100 (0.838–1.445)
Animal food consumed (1)	0.589	0.958 (0.819–1.118)
Attitudes to vaccination (2)	0.572	0.987 (0.942–1.033)
Attitudes to animal use (3)	0.102	0.975 (0.942–1.005)
Importance of animal welfare	0.724	1.017 (0.927–1.115)
Attitudes to antibiotic use in livestock	0.191	1.022 (0.989-1.005)
Knowledge of antibiotic resistance (5) *	0.002 *	1.116 (1.040–1.199)
Agreement with “I am very concerned about the issue of antibiotic resistance but not sure what I can do” *	<0.001 *	0.728 (0.632–0.836)
Are you afraid that antibiotic resistance might affect you one day?—yes *	0.012 *	0.652 (0.466–0.909)
Importance of AMR	0.889	0.992 (0.888–1.108)
Antibiotic use in last 12 months	0.360	1.148 (0.855–1.544)
Experience of AMR in family *	0.036 *	0.703 (0.505 –0.978)
Perceived risk of antibiotics *	<0.001 *	0.729 (0.648–0.819)
Awareness of AMR *	0.018 *	0. 798 (0.662–0.961)
Importance of sustainability	0.986	1.001 (0.893–1.121)

**Table 5:** Results of ordinal logistic regression model for consumer preferences.

Variable	*p*-Value	Odds Ratio (95% Confidence Interval)
Being female	0.004	1.61 (1.16-2.22)
Having had a family member affected by antibiotic failure	0.019	0.67 (0.47–0.93)
Knowledge of antibiotic use and resistance	0.007	1.12 (1.03–1.21)
Being concerned about AMR, but not knowing what to do about it	0.002	0.78 (0.67–0.91)
Perceived risk of antibiotic use in livestock	<0.001	0.78 (0.68–0.90)

**Table 6:** Predictor variables.

Gender
Age
Urban/rural location
Keeping pets and/or livestock
Animal products consumed (1)
Attitudes to vaccination (2)
Attitudes to animal use (3)
Importance of animal welfare
Attitudes to antibiotic use in livestock (4)
Knowledge of antibiotic resistance (5)
Agreement with “I am very concerned about the issue of antibiotic resistance but not sure what I can do”
Fear of being affected personally by AMR
Importance of AMR
Antibiotic use in last 12 months
Experience of antibiotic treatment failure in self or family member
Perceived risk of antibiotics
Awareness of antibiotic resistance
Importance of sustainability

## Data Availability

The datasets used for analysis were kindly shared by the participating supermarkets with the authors as academic partners in the Food Industry Initiative on Antimicrobial Resistance working group, but permission has not been granted for further data sharing.

## References

[1] Walsh F. (2013). Antibiotics Resistance ‘As Big As Terrorism’—Medical Chief. BBC News [Online]. https://www.bbc.co.uk/news/health-21737844.

[2] ESPAUR Report 2021 to 2022 English Surveillance Programme for Antimicrobial Utilisation and Resistance (ESPAUR) Report 2021 to 2022, London: UK Health Security Agency; November 2022. https://www.gov.uk/government/news/new-antibiotic-resistant-infections-rise-to-178-per-day-in-england.

[3] Fischer M.M., Bild M. (2019). Hospital use of antibiotics as the main driver of infections with antibiotic-resistant bacteria—A reanalysis of recent data from the European Union. bioRxiv.

[4] Chang Q., Wang W., Regev-Yochay G., Lispitch M., Hanage W. (2015). Antibiotics in agriculture and the risk to human health: How worried should we be?. Evol. Appl..

[5] Magouras I., Carmo L.P., Stärk K.D.C., Schüpbach-Regula G. (2017). Antimicrobial Usage and -Resistance in Livestock: Where Should We Focus?. Front. Vet. Sci..

[6] Bennani H., Mateys A., Mays N., Eastmure E., Stãrk K.D.C., Häsler B. (2020). Review of antimicrobial use and antimicrobial resistance in the food chain. Antibiotics.

[7] Review of Antimicrobial Resistance (2016). Tackling Drug-Resistant Infections Globally: Final Report. The Review on Antimicrobial Resistance.

[8] (2021). UK-VARSS Veterinary Antibiotic Resistance and Sales Surveillance Report (UK-VARSS 2021). New Haw, Addlestone: Veterinary Medicine Directorate. www.gov.uk.

[9] Hudson J.A., Frewer L.J., Jones G., Brereton P., Whittingham M.J., Stewart G. (2017). The Agri-food chain and antimicrobial resistance: A review. Trends Food Sci. Technol..

[10] Lechner I., Freivogel C., Stärk K.D.C., Visschers V.H.M. (2020). Exposure Pathways to Antimicrobial Resistance at the Human-Animal Interface—A Qualitative Comparison of Swiss Expert and Consumer Opinions. Front. Public Health.

[11] Hocknell S., Hughes A., Roe E., Keevil B., Wrigley N., Lowe M. (2018). Corporate Food Retailers, Meat Supply Chains, and the Responsibilities of Tackling Antimicrobial Resistance. Stakeholder Report. https://eprints.ncl.ac.uk/file_store/production/253101/D0EA9E6C-A2E1-4596-89BF-AC4B6A7E556E.pdf.

[12] Doidge C., Hudson C., Lovatt F., Kalevr J. (2019). To prescribe or not to prescribe? A factorial survey to explore veterinarians’ decision making when prescribing antimicrobials to sheep and beef farmers in the UK. PLoS ONE.

[13] Fischer K., Sjöström K., Stienström A., Emanuelson U. (2019). Dairy farmers’ perspectives on antibiotic use: A qualitative study. J. Dairy Sci..

[14] Adam K. (2019). Balancing conflicting demands: Factors influencing vets’ choice of antimicrobial agent. Vet. Rec..

[15] Schneider S., Salm F., Vincze S., Moeser A., Petruschke I., Schmücker K., Ludwig N., Hanke R., Schröder C., Gropmann A. (2018). Perceptions and attitudes regarding antibiotic resistance in Germany: A cross-sectoral survey amongst physicians, veterinarians, farmers and the general public. J. Antimicrob. Chemother..

[16] Begemann S., Watkins F., van Hoyweghen I., Vivancos R., Christley R., Perkins E. (2020). The Governance of UK Dairy Antibiotic Use: Industry-Led Policy in Action. Front. Vet. Sci..

[17] Alban L., Dahl J., Andreasen M., Petersen J.V., Sandberg M. (2013). Possible impact of the “yellow card” antimicrobial scheme on meat inspection lesions in Danish finisher pigs. Prev. Vet. Med..

[18] Eriksen E.O., Smed S., Klit K.J., Olsen J.E. (2019). Factors influencing Danish veterinarians’ choice of antimicrobials prescribed for intestinal diseases in weaner pigs. Vet. Rec..

[19] Carmo L.P., Nielsen L.R., Alban L., da Costa P.M., Schüpbach-Regula G., Magoulras I. (2018). Veterinary Expert Opinion on Potential Drivers and Opportunities for Changing Antimicrobial Usage Practices in Livestock in Denmark, Portugal, and Switzerland. Front. Vet. Sci..

[20] Hinchliffe S., Butcher A., Rahman M.M. (2018). The AMR problem: Demanding economies, biological margins, and co-producing alternative strategies. Palgrave Commun..

[21] McCullough A.R., Parekh S., Rathbone J., Del Mar C.B., Hoffman T.C. (2016). A systematic review of the public’s knowledge and beliefs about antibiotic resistance. J. Antimicrob. Chemother..

[22] Regan A., Sweeney S., McKernan C., Benson T., Dean M. (2022). Consumer perception and understanding of the risks of antibiotic use and antimicrobial resistance in farming. Agric. Hum. Values.

[23] Cornejo J., Cabezón C., San Martín B., Lapierre L. (2018). Assessment of Consumer Perceptions on the Use of Antimicrobials in Production Animals in Chlle. J. Food Prot..

[24] Ding Q., Gao J., Ding X., Huang D., Zhao Y., Yang M. (2022). Consumers’ knowledge, attitude, and behaviour towards antimicrobial resistance and antimicrobial use in food production in China. Front. Public Health.

[25] Wemette M., Greiner Safi A., Wolverton A.K., Beuvais W., Shapiro M., Moroni P., Welcome F.L., Ivanek R. (2021). Public perceptions of antibiotic use on dairy farms in the United States. J. Dairy Sci..

[26] Busch G., Kassas B., Palma M.A., Risius A. (2020). Perceptions of antibiotic use in livestock farming in Germany, Italy and the United States. Livest. Sci..

[27] Goddard E., Hartmann M., Klink-Lehmann J. (2017). Public Acceptance of Antibiotic Use in Livestock Production Canada and Germany. Proc. Food Syst. Dyn..

[28] Bradford H., McKernan C., Elliott C., Dean M. (2022). Consumers’ perceptions and willingness to purchase pork labelled ‘raised without antibiotics. Appetite.

[29] Barret J.R., Innes G.K., Johnson K.A., Lhermie G., Ivanek R., Safi A.G., Lansing D. (2021). Consumer perceptions of antimicrobial use in animal husbandry: A scoping review. PLoS ONE.

[30] Meerza S.I.A., Gulab S., Brooks K.R., Gustafson C.R., Yiannaka A.U.S. (2022). Consumer Attitudes towards Antibiotic Use in Livestock Production. Sustainability.

[31] Kirchelle C. (2018). Pharming animals: A global history of antibiotics in food production (1935–2017). Palgrave Commun..

[32] Woods A. (2018). Decentring antibiotics: UK responses to the diseases of intensive pig production (ca. 1925-65). Palgrave Commun..

[33] Regulation (EU) 2019/6 of the European Parliament and of the Council of 11 December 2018 on Veterinary Medicinal Products and Repealing Directive 2001/82/EEC. https://eur-lex.europa.eu/legal-content/EN/AUTO/?uri=celex:32019R0006.

[34] Cervantes H.M. (2015). Antibiotic-free poultry production: Is it sustainable?. J. Appl. Poult. Res..

[35] Hughes A., Roe E., Hocknell S. (2021). Food supply chains and the antimicrobial resistance challenge: On the framing, accomplishments and limitations of corporate responsibility. Environ. Plan. A Econ. Space.

[36] Jia S., McWhorter A.R., Andrews D.M., Underwood G.J., Chousalkar K.K. (2020). Challenges in vaccinating layer hens against Salmonella TyphimuriumI. Vaccines.

[37] Redding L.E., Parsons B., Bender J.S. (2021). Educational interventions to address misconceptions about antibiotic residues in milk can alter consumer perception and may affect purchasing habits. J. Dairy Sci..

[38] Schell R.C., Bulut E., Padda H., Safi A.G., Moroni P., Ivanek R. (2022). Responsible antibiotic use labeling and consumers’ willingness to buy and pay for fluid milk. J. Dairy Sci..

[39] Ancilotti M., Nilsson E., Nordvall A.-C., Oljans E. (2022). The Status Quo problem and the Role of Consumers against Antimicrobial Resistance. Front. Sustain. Food Syst..

[40] Scannell J.W., Bruce A. (2015). Antibiotics: Expect to use less, more responsibly. Vet. Rec..

[41] Roselius T. (1971). Consumer Rankings of Risk Reduction Methods. J. Mark..

[42] R Core Team (2019). R: A Language and Environment for Statistical Computing.

